# A Compend of Electricity

**Published:** 1887-05

**Authors:** 


A COMPEND OF ELECTRICITY AND ITS MEDICAL AND SURGICAL USES. *By* CHARLES F. MASON, M.D. *With an introduction by* CHARLES H. MAY, M.D. *12 mo. pp., xx and 108. Philadelphia:* P. BLAKISTON, SON & CO. *Chicago:* W. T. KEENER. *1887.*

This is a small book; and under the conditions imposed it cannot be entirely satisfactory, because of the great condensation. However, the chapters on "Electro-therapeutics," and "Electricity in Surgery," which are the most practical parts of the work, are very well done. The class to which this volume belongs can scarcely be commended, because, no matter how well they may be prepared, the tendency is to cultivate superficial knowledge. As quiz-compends they are good, but as manuals for the general practitioner of medicine they are not.

